# Use of IP-10 detection in dried plasma spots for latent tuberculosis infection diagnosis in contacts via mail

**DOI:** 10.1038/s41598-019-40778-1

**Published:** 2019-03-08

**Authors:** R. Villar-Hernández, I. Latorre, M. L. De Souza-Galvão, M. A. Jiménez, J. Ruiz-Manzano, J. Pilarte, E. García-García, B. Muriel-Moreno, A. Cantos, N. Altet, J. P. Millet, Y. González-Díaz, I. Molina-Pinargote, C. Prat, M. Ruhwald, J. Domínguez

**Affiliations:** 1 Servei de Microbiología, Hospital Universitari Germans Trias i Pujol, Institut d’Investigació Germans Trias i Pujol, Carretera del Canyet, 08916 Badalona, Barcelona, Spain; 2 CIBER Enfermedades Respiratorias, CIBERES, Instituto de Salud Carlos III, Carretera del Canyet, 08916 Badalona, Barcelona, Spain; 3 Universitat Autònoma de Barcelona, Carretera del Canyet, 08916 Badalona, Barcelona, Spain; 4 Unitat de Tuberculosi de Drassanes, Hospital Universitari Vall d’Hebron. Av. de les Drassanes, 17, 08001 Barcelona, Barcelona, Spain; 5 Servei de Pneumologia, Hospital Universitari Germans Trias i Pujol, Carretera del Canyet, 08916 Badalona, Barcelona, Spain; 6 Unidad Clínica de Tratamiento Directamente Observado “Serveis Clinics”, Carrer de García Mariño, 4, 08022 Barcelona, Spain; 7 CIBER de Epidemiología y Salud Pública, CIBEREESP, Instituto de Salud Carlos III, Carretera del Canyet, 08916 Badalona, Barcelona, Spain; 8 Department of Infectious Disease Immunology Statens Serum Institut, Copenhagen, Denmark - Artillerivej 5, 2300 Copenhagen, Denmark

## Abstract

The aim of this study was to test the use of IP-10 detection in dried plasma from contact studies individuals (contacts of smear positive patients), by comparing it with IP-10 and IFN-γ detection in direct plasma, to establish IP-10 detection in DPS as a useful assay for LTBI diagnosis. Whole blood samples were collected from 80 subjects: 12 with active tuberculosis (TB), and 68 from contact studies. The amount of IFN-γ produced by sensitized T cells was determined in direct plasma by QuantiFERON Gold In-Tube test. IP-10 levels were determined in direct and dried plasma by an *in-house* ELISA. For dried plasma IP-10 determination, two 25 µl plasma drops were dried in Whatman903 filter paper and sent by mail to the laboratory. Regarding TB patients, 100.0%, 91.7% and 75.0% were positive for IFN-γ detection and IP-10 detection in direct and dried plasma, respectively. In contacts, 69.1%, 60.3% and 48.5% had positive results after IFN-γ and IP-10 in direct and dried plasma, respectively. The agreement among *in vitro* tests was substantial and IP-10 levels in direct and dried plasma were strongly correlated (r = 0.897). In conclusion, IP-10 detection in dried plasma is a simple and safe method that would help improve LTBI management.

## Introduction

Tuberculosis (TB) still causes high morbidity and mortality rates worldwide. The key to disease control is early diagnosis and efficient treatment regimens^1^. Latent tuberculosis infection (LTBI) screening is traditionally based on the tuberculin skin test (TST). However, this test is limited by cross-reaction with the *Mycobacterium bovis* strain present in the bacilli Calmette-Guérin (BCG) vaccine and non-tuberculous mycobacteria (NTM)^2^. Interferon (IFN)-γ release assays (IGRAs), which measure T-cell-mediated responses after specific *Mycobacterium tuberculosis* antigen stimulation either from whole blood (QuantiFERON technology; QFN, Qiagen, Düsseldorf, Germany) or peripheral blood lymphocytes (T-SPOT.TB; Oxford Immunotec Limited, Abingdon, UK)^3–5^, have appeared as an alternative for the TST. Both TST and IGRAs provide indirect evidence of the presence of the bacilli by measuring cell sensitization. Despite the fact that IGRAs positive predictive value has been shown to be poor^6^, their negative predictive value is excellent and correlates better with contacts degree of *M. tuberculosis* exposure that the TST^7^.

In the past few years, the use of IFN-γ inducible protein 10 (IP-10) has been studied as a biomarker of LTBI. This chemokine is largely expressed by antigen-presenting cells previously induced by innate and adaptive mechanisms. During the adaptive immune response, IP-10 production is mainly driven by T-cell-derived IFN-γ^8,9^. IP-10 levels are reported to be 100-fold of those of IFN-γ, suggesting that it can be a promising diagnostic marker in TB^9,10^, as well as a good option for assay simplification and miniaturization such as lateral flow, dried blood spots and molecular detection^9,11^. IP-10 detection has been proven more robust than IFN-γ in HIV-infected patients and young children^12–17^.

Having procedures that make sample transportation from the extraction/collection sites to the laboratory simple and safe, and that also detect infection markers other than IFN-γ, would be of use, easing IGRA implementation, and improving LTBI control. An example of these procedures, is the dried blood and plasma drops. The use of dried blood in diagnostics was introduced for metabolic diseases in neonatal population in 1963. Since then, this technique has had different applications both with dried blood and plasma, including TB infection diagnosis and management^18–21^. Blauenfeldt *et al*. found that IP-10 mRNA isolated from dried blood spots and IP-10 isolated from dried plasma spots (DPS) had higher levels in active TB patients compared to uninfected controls^20^. Hence, the detection of IP-10 in DPS has also been described as a possible indicator of anti-TB treatment efficacy, as it declines in response to anti-TB chemotherapy^19^. Regarding LTBI diagnosis, we previously described the stability of IP-10 in DPS from active TB patients and non-infected individuals, showing that, IP-10 levels detected in DPS are stable and comparable to those detected in direct plasma^18^. However, there is little experience addressing IP-10 detection in DPS in contacts^22^ yielding a low number of patients (2/60, 3.3%) with positive tests results.

Due to this knowledge gap, the main objective of this study is to test the use of IP-10 detection in dried plasma from contact studies individuals (contacts of smear positive patients), by comparing it with IP-10 and IFN-γ detection in direct plasma, to establish IP-10 detection in DPS as a useful assay for LTBI diagnosis.

## Results

### Study population

A total of 80 subjects were included in this study. Twelve of them (15.0%) were patients with microbiologically confirmed pulmonary active TB and 68 (85.0%) were individuals from contact tracing studies. TST was performed in 69 patients (86.3%); all of them positive, except one that was also negative for QuantiFERON Gold In-Tube (QFN-G-IT). This is due to the fact that the unit, where the samples come from, acts as a reference centre of the area and mainly receives TST positive patients from contact studies for further evaluation. Demographic and clinical data is shown in Table 1. For further analysis purposes we grouped the patients in active TB, LTBI and uninfected controls. We considered as LTBI patients those contacts with a positive QFN-G-IT result (69.1%; 47/68) and as uninfected controls those with a negative QFN-G-IT result (30.9%; 21/68). Furthermore, we also grouped contacts in three groups based on the exposure degree.

**Table 1:** Demographic characteristics of all patients included in the study.

Variable	Overall patients (%) *n* = 80	Active TB patients (%) *n* = 12	Contacts (%) *n* = 68
Age, mean (years) ± SD	29.5 ± 11.6	33.5 ± 9.2	28.8 ± 11.9
Gender			
Male	39 (48.8)	10 (83.3)	29 (42.6)
Female	41 (51.2)	2 (16.6)	39 (57.4)
Country of birth			
High TB incidence	32 (40.0)	8 (66.7)	24 (35.3)
Low TB incidence	43 (53.8)	4 (33.3)	39 (57.3)
Unknown	5 (6.2)	0 (0.0)	5 (7.4)
BCG			
Yes	28 (35.0)	2 (16.6)	26 (38.2)
No	35 (43.7)	5 (41.7)	30 (44.1)
Unknown	17 (21.3)	5 (41.7)	12 (17.6)
QFN-G-IT			
Positive	59 (73.8)	12 (100.0)	47 (69.1)
Negative	21 (26.2)	0 (0.0)	21 (30.9)

### Cytokine levels detected

**Figure 1:**
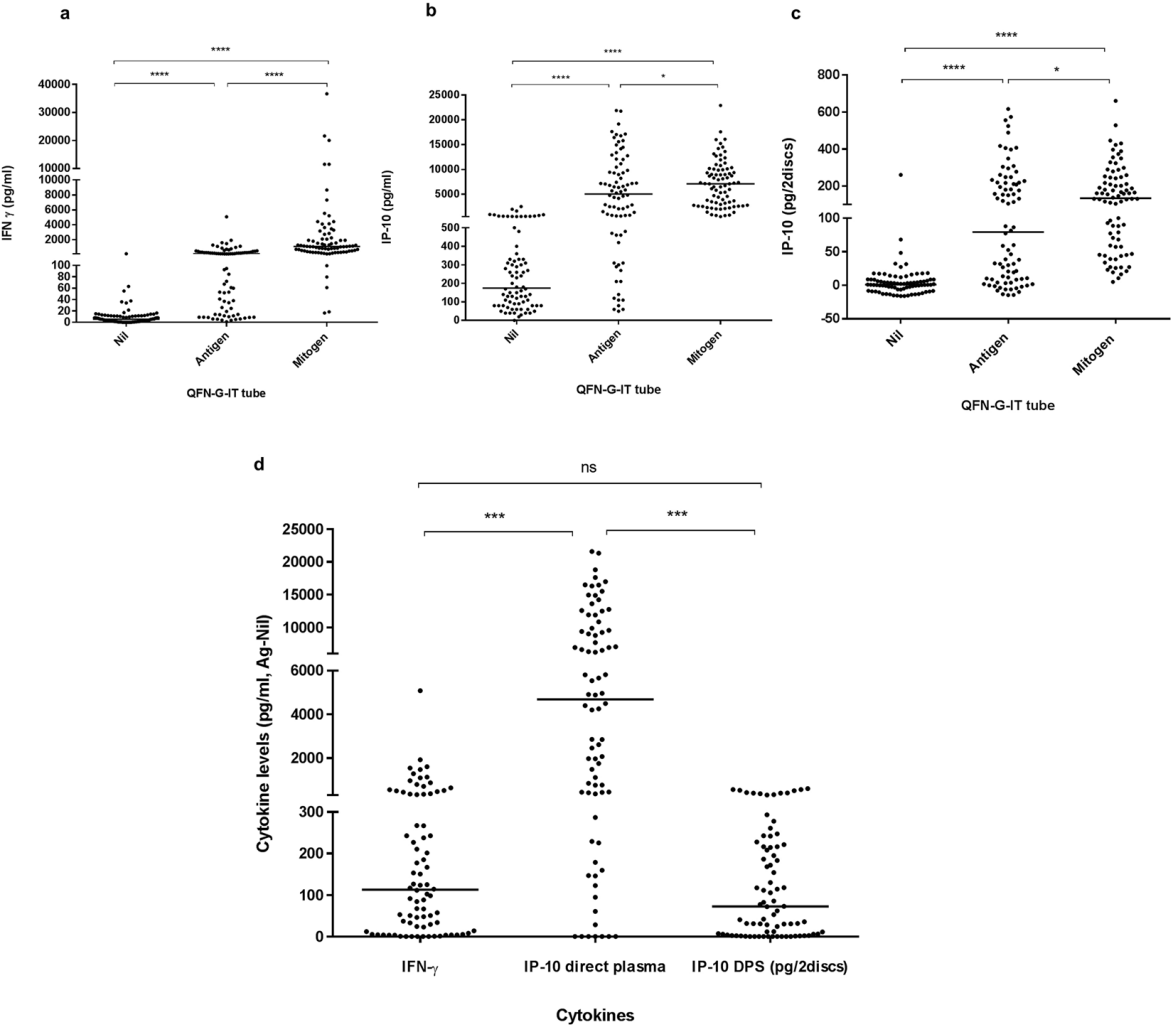
IFN-γ levels in plasma (**a**) and IP-10 measured both in direct plasma (**b**) and DPS (**c**) after nil, antigen and mitogen stimulation, and (**d**) comparison between IFN-γ and IP-10 levels after antigen-specific stimulation. The median cytokine levels are represented by a horizontal line. Cytokine levels in mitogen stimulated samples are higher than antigen and nil stimulated ones in every method used. The median cytokine levels are represented by a horizontal line. Differences in the cytokine levels between groups were analyzed using Mann-Whitney U test (ns = p > 0.05, *p ≤ 0.05 ***p ≤ 0.0001).

**Figure 2:**
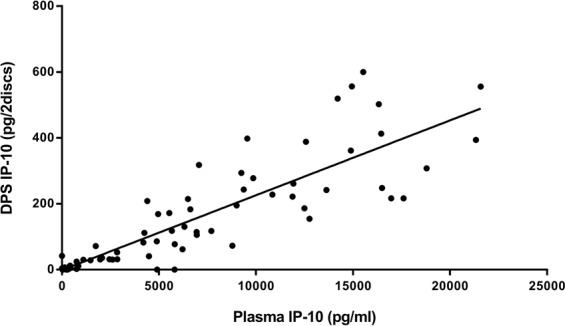
Correlation of IP-10 detected in dried plasma spots (DPS) and direct plasma. IP-10 detection in nil, antigen and mitogen stimulated samples. Correlation assessed using Spearman correlation coefficient (r = 0.897, p < 0.0001).

**Figure 3:**
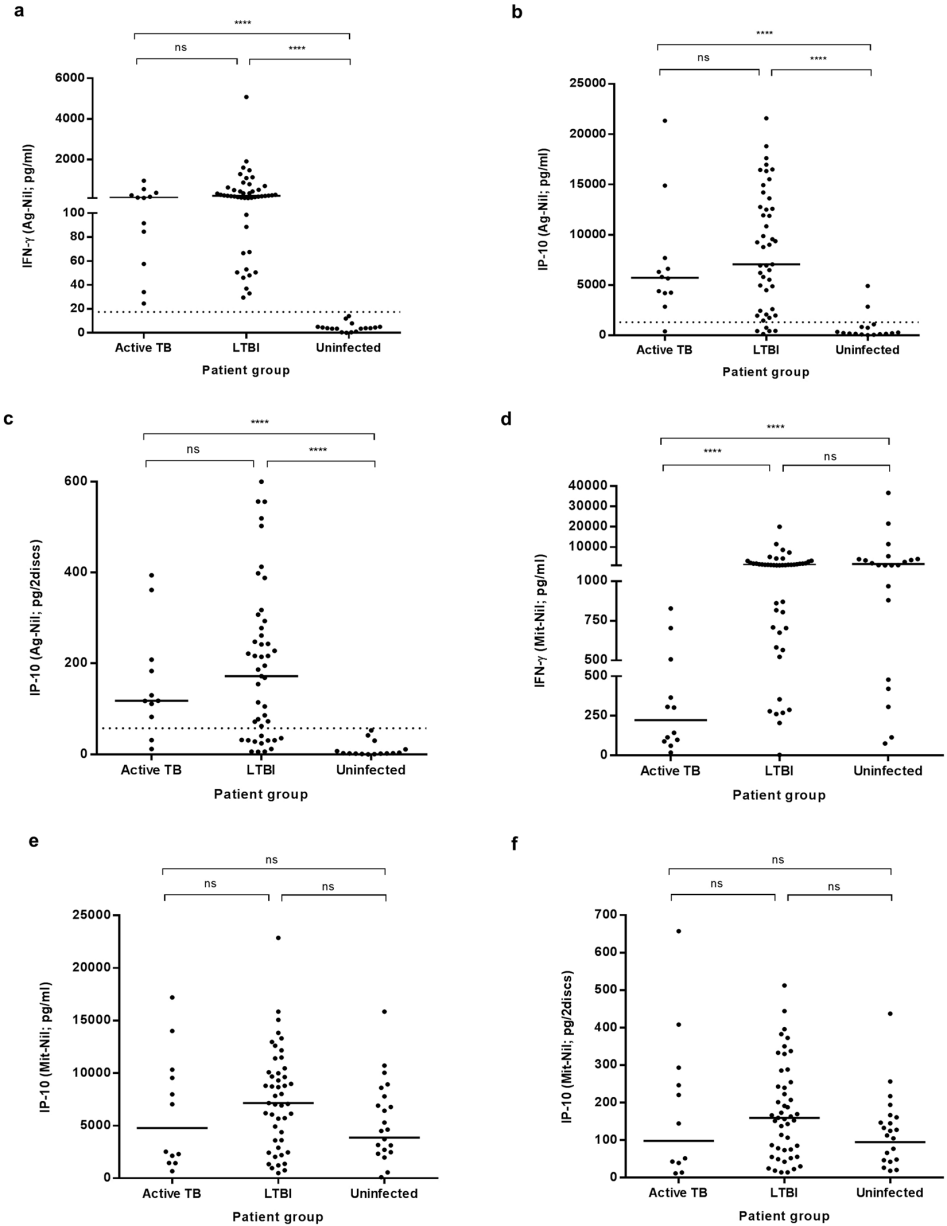
Cytokine levels per study group after antigen and mitogen stimulation. Specific antigen stimulation: (**a**) IFN-γ levels in active TB patients (median = 119 pg/ml; interquartile range (IQR): 64.3–331.9), LTBI patients (median = 216.5 pg/ml; IQR: 98.5–515.0) and uninfected controls (median = 3.5 pg/ml; IQR: 0.3–4.8), (**b**) IP-10 levels in direct plasma samples in active TB patients (median = 5735 pg/ml; IQR: 4213–7428), LTBI patients (median = 7060 pg/ml; IQR: 2060–12760) and uninfected controls (median = 160 pg/ml; IQR: 15–555), and (**c**) IP-10 levels in DPS in active TB patients (median = 117.9 pg/2discs; IQR: 44.1–202.0), LTBI patients (median = 172 pg/2discs; IQR: 31.8–277.8) and uninfected controls (median = 1.8 pg/2discs; IQR: −0.4–5.5). Mitogen stimulation: (**d**) IFN-γ levels in active TB patients (median = 223 pg/ml; IQR: 91.9–471.8), LTBI patients (median = 1273 pg/ml; IQR: 704.5–2079.0) and uninfected controls (median = 1161 pg/ml; IQR: 679.5–4110.0), (**e**) IP-10 levels in direct plasma samples in active TB patients (median = 4785 pg/ml; IQR: 1623–10133), LTBI patients (median = 7160 pg/ml; IQR: 3610–10080) and uninfected controls (median = 4630 pg/ml; IQR: 2600–8195), and (**f**) IP-10 levels in DPS in active TB patients (median = 97.8 pg/2discs; IQR: 19.9–281.5), LTBI patients (median = 159.3 pg/2discs; IQR: 73.0–254.7) and uninfected controls (median = 124.5 pg/2discs; IQR: 47.3–163.8). Uninfected controls are contacts with negative QFT. Statistical analysis was performed using Mann-Whitney U test (ns = p > 0.05, ****p ≤ 0.0001). Dotted line indicates Ag-Nil positivity cut-off: IFN-γ = 17.5 pg/ml, IP-10 direct plasma = 1300 pg/ml, IP-10 DPS = 57.4 pg/2discs.

We compared the cytokines (IFN-γ and IP-10) levels detected (i) among each QFN-G-IT tubes (nil, antigen and mitogen), (ii) by each *in vitro* test, and (iii) in each patient group (active TB, LTBI and uninfected controls). The amount of IFN-γ and IP-10 detected in each QFN-G-IT test tube was significantly different (*p* < 0.05) between tubes being the highest amount present in the mitogen tube and the lowest in the nil, as expected (Fig. 1a–c). The amount of IP-10 detected in direct plasma was significantly higher (*p* < 0.0001) than that of DPS and IFN-γ with a median value of 4686 pg/ml (IQR = 411.2–9785), 72.4 pg/2discs (IQR = 5.6–220.4), and 113.0 pg/ml (IQR = 16.3–342.4), respectively, when subtracting the nil from the antigen stimulated tube (Fig. 1d). Moreover, IP-10 levels in direct plasma correlated well with those in DPS (Spearman correlation coefficient r = 0.897, *p* < 0.0001) (Fig. 2), meaning that both tests are comparable and that IP-10 is stable in DPS. After stimulation with specific antigens, LTBI patients had a higher production of cytokines than TB patients (not statistically significant) and uninfected controls (*p* < 0.0001) (Fig. 3a–c). After mitogen stimulation, LTBI patients had higher IFN-γ and IP-10 levels than active TB patients (only significantly different (*p* < 0.0001) when looking at IFN-γ levels) and uninfected controls (not significant) (Fig. 3d–f).

### ROC curve analysis

**Figure 4:**
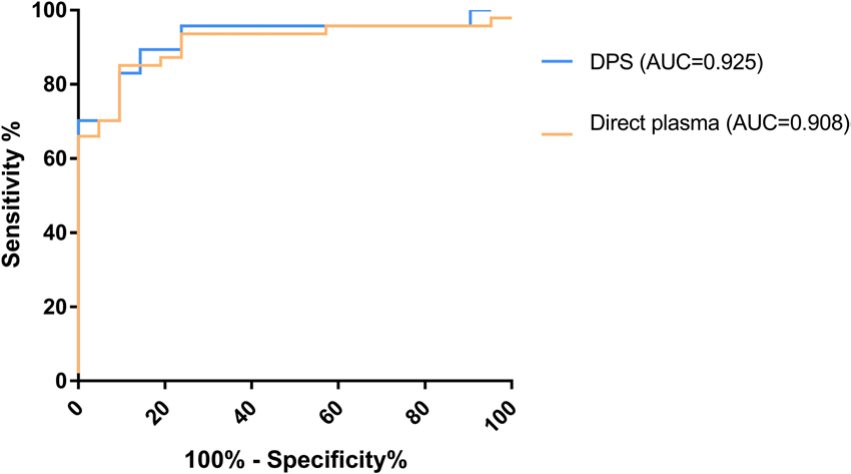
Receiver operating characteristic (ROC) curve analysis. Antigen-specific (ag-nil) release of IP-10 in DPS and direct plasma samples. Areas under the curve (AUCs) were comparable using both methods: DPS AUC (blue line) = 0.925 and direct plasma AUC (orange line) = 0.908. QFN-G-IT positive contacts were considered as LTBI patients and QFN-G-IT negative contacts as uninfected controls.

In order to establish positivity cut-offs for IP-10 detection, we performed ROC curve analysis. The areas under curves (AUC), of both IP-10 detection tests were comparable (AUC_direct plasma_ = 0.908 and AUC_DPS_ = 0.925). After analysing the AUCs, we chose the following cut-offs: 1.3 ng/ml (sensitivity 83.7%, 95% confidence interval [CI] 69.3–93.2; specificity 94.1%, 95% CI 71.3–99.8) in direct plasma detection and 57.4 pg/2discs (sensitivity 67.4%, 95% CI 51.5–80.9; specificity 100%, 95% CI 80.5–100.0) in DPS (Fig. 4). Thus, every value above 1.3 ng/ml in direct plasma and 57.4 pg/2discs in DPS, was considered positive. As indeterminate result cut-offs, we chose: 0.1 ng/ml for the mitogen in direct plasma detection, and 17.8 pg/2discs in DPS. Therefore, results considered negative using the positivity cut-off but with cytokine production lower than 0.1 ng/ml (for direct plasma) and 17.8 pg/2discs (for DPS) after mitogen stimulation and nil subtraction, were considered indeterminate.

### Test results

Active TB patients had TST performed in 58.3% (7/12) of cases obtaining 100.0% (7/7) positive results, and contacts had 91.2% (62/68) TST performed, yielding 98.4% (61/62) positive results (Table 2).

**Table 2:** Results (%) obtained with each *in vitro* technique in each group of patients.

Patient classification	TST	QFN-G-IT	IP-10 direct plasma^a^	IP-10 DPS^b^
Active TB (*n* = 12)				
Positive	7 (100.0)	12 (100.0)	11 (91.7)	9 (75.0)
Negative	0 (0.0)	0 (0.0)	1 (8.3)	0 (0.0)
Indeterminate^a^	—	0 (0.0)	0 (0.0)	3 (25.0)
Contacts (*n* = 68)				
Positive	61 (98.4)	47 (69.1)	41 (60.3)	33 (48.5)
Negative	1 (1.6)	21 (30.9)	27 (39.7)	32 (47.1)
Indeterminate^a^	—	0 (0.0)	0 (0.0)	3 (4.4)
*LTBI (n* = *47)*				
Positive	41 (100.0)	47 (100.0.0)	39 (83.0)	33 (70.2)
Negative	0 (0.0)	0 (0.0)	8 (17.0)	11 (23.4)
Indeterminate^a^	—	0 (0.0)	0 (0.0)	3 (6.4)
*UC (n* = *21)*				
Positive	20 (95.2)	0 (0.0)	2 (9.5)	0 (0.0)
Negative	1 (4.8)	21 (100.0)	19 (90.5)	21 (100.0)
Indeterminate^a^	—	0 (0.0)	0 (0.0)	0 (0.0)
Overall (*n* = 80)				
Positive	68 (98.6)	59 (73.8)	52 (65.0)	42 (52.5)
Negative	1 (1.4)	21 (26.2)	28 (35.0)	32 (40.0)
Indeterminate^a^	—	0 (0.0)	0 (0.0)	6 (7.5)

^a^Cut-offs: Ag-Nil = 1.3 ng/ml, Mit-Nil = 0.1 ng/ml. ^b^Cut-offs: Ag-Nil = 57.4 pg/2discs, Mit-Nil = 17.8 pg/2discs. UC: uninfected controls.

Regarding the QFN-G-IT test overall, 73.8% (59/80) of the samples had a positive result, 26.2% (21/80) had a negative result and none of the patients had an indeterminate result (Table 2). All TB patients had a positive QFN-G-IT. From the contacts with positive QFN-G-IT (69.1%, 47/68), the 41 that had the TST done were also positive for this test. From the contacts with negative QFN-G-IT (30.9%, 21/68), 4.8% (1/21) was also negative by TST, but the remaining 95.2% (20/21) were positive (11 out of these 20, 55.0%, were BCG vaccinated and the average induration size of non-BCG vaccinated was 9 mm).

**Figure 5:**
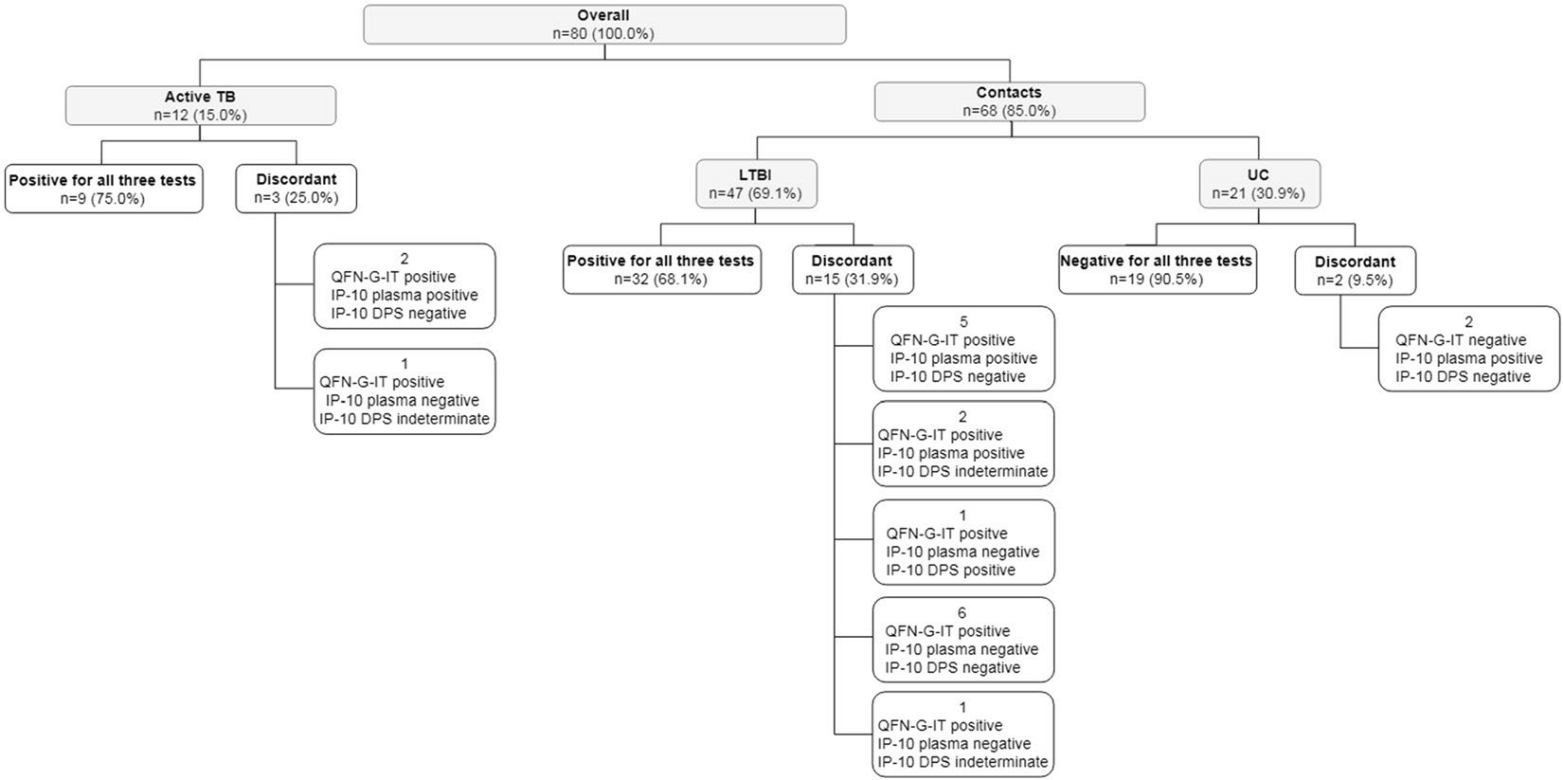
Flowchart of participants and test result. Graphical representation of the participants included and the results obtained for each test (QFN-G-IT, IP-10 detection in direct plasma and IP-10 detection in DPS) using the selected cut-offs. UC: uninfected controls.

Using the selected cut-offs for positive and indeterminate results, the percentages of overall positivity in direct plasma and DPS were 65.0% (52/80) and 52.5% (42/80), respectively (Table 2, Fig. 5). IP-10 detection in direct plasma yielded a 35.0% (28/80) of negative results and no indeterminate resutls. IP-10 detection in DPS yielded a 40.0% (32/80) of negative results and a 7.5% (6/80) of indeterminate results. Not all samples from active TB patients had a positive IP-10 result: 8.3% (1/12) was negative for IP-10 detection in direct plasma but indeterminate in DPS, and other 2 (3/12, a total of 25.0%) were indeterminate when using DPS. Regarding contacts, the amount of positive and negative results obtained by IP-10 detection in direct plasma is distributed similarly to that of QFN-G-IT. IP-10 detection in DPS in contacts also yielded both positive and negative results, but also 4.4% (3/68) were indeterminate which were positive for QFN-G-IT (Table 2, Fig. 5).

A 6.2% of the contact individuals (5/80) from low prevalence countries were not BCG vaccinated but had a positive TST, a negative QFN-G-IT and IP-10 in DPS result, and 4 of them had also a negative result in IP-10 detection in direct plasma. The agreement between IP-10 detection in plasma and in DPS was strong (κ = 0.77), as that of the QFN-G-IT test and IP-10 detection in direct and DPS (κ = 0.68 in both cases). Combination of both cytokines detection in direct plasma from our total study population yielded a 76.0% (61/80) of positive results. This means a 2.5% increase in the number of positive results compared to those obtained by QFN-G-IT alone and 11.25% compared to IP-10 detection alone. Regarding contacts, cytokine detection in direct plasma yielded a 72.0% (49/68) of positive results. This is a 3.0% increase in the number of positive results compared to those obtained by QFN-G-IT alone and a 12.0% compared to IP-10 detection alone.

### Contact groups

**Figure 6:**
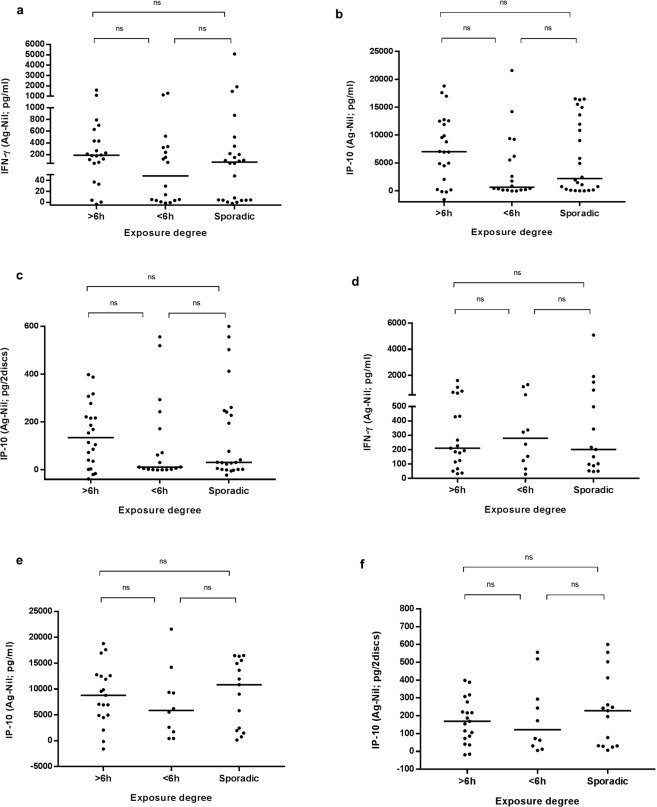
Cytokine levels in overall contacts (**a**–**c**) and in LTBI contacts (**d**–**f**) regarding the exposure degree. (**a**) IFN-γ detected by QFN-G-IT in direct plasma (pg/ml). Median values: 189 pg/ml, 48 pg/ml, 70.75 pg/ml in >6 h, <6 h and sporadic contatcs, respectively. (**b**) IP-10 detected in direct plasma (pg/ml). Median values: 7003 pg/ml, 640 pg/ml and 2206 pg/ml in >6 h, <6 h and sporadic contatcs, respectively. (**c**) IP-10 detected in DPS (pg/2discs). Median values: 134.5 pg/2discs, 11.6 pg/2discs and 31.45 pg/2discs in >6 h, <6 h and sporadic contatcs, respectively. (**d**) IFN-γ detected by QFN-G-IT in direct plasma (pg/ml). Median values: 210.5 pg/ml, 280 pg/ml, 201.5 pg/ml in >6 h, <6 h and sporadic contatcs, respectively. (**e**) IP-10 detected in direct plasma (pg/ml). Median values: 8780 pg/ml, 5874 pg/ml and 10841 pg/ml in >6 h, <6 h and sporadic contatcs, respectively. (**f**) IP-10 detected in DPS (pg/2discs). Median values: 168.6 pg/2discs, 122 pg/2discs and 227.8 pg/2discs in >6 h, <6 h and sporadic contatcs, respectively. Statistical analysis was performed using Mann-Whitney U test (ns = p > 0.05). Four contacts had unknown exposure degree (data not shown).

In order to study more in depth contact individuals, we grouped them regarding the contact degree: more than 6 hours (22/64, 34.4%), less than 6 hours (18/64, 28.1%) and sporadic contacts (24/64, 37.5%) (four contacts are excluded as the exposure degree is unknown) (Table 3). Although it is not statistically significant, contacts with less than 6 hours of exposure are the group with more negative results by the three *in-vitro* tests; meanwhile contacts with an exposure above 6 hours have the majority of positive results. IFN-γ in direct plasma, and IP-10 levels, both in direct and dried plasma, among the three groups were similar (no statistically significant differences among them, *p* > 0.05) but higher in more than 6-hour contacts (Fig. 6a–c). Regarding LTBI individuals (positive QFN-G-IT result) with known exposure degree, 43.2% (19/44) were more than 6-hour contacts, 22.7% (10/44) were less than 6-hour contacts and 34.1% (15/44) were sporadic contacts. As well as when considering the total contact cohort, LTBI individuals with less than 6 hours of exposure have more negative IP-10 test results, and those with more than 6 hours of exposure have more positive results (Table 3). Although IFN-γ levels in these patients are higher in less than 6-hour contacts, and IP-10 levels are higher in sporadic contacts, the differences among groups remains as not statistically significant (*p* > 0.05) (Fig. 6d–f).

**Table 3:** Contact test results (%) per exposure degree.

Exposure time	QFN-G-IT	IP-10 direct plasma	IP-10 DPS
>6 h (*n* = 22)			
Positive	19 (86.4)	17 (77.3)	15 (68.2)
Negative	3 (13.6)	5 (22.7)	6 (27.3)
Indeterminate	0 (0.0)	0 (0.0)	1 (4.5)
<6 h (*n* = 18)			
Positive	10 (55.6)	7 (38.9)	7 (38.9)
Negative	8 (44.4)	11 (61.1)	11 (61.1)
Indeterminate	0 (0.0)	0 (0.0)	0 (0.0)
Sporadic (*n* = 24)			
Positive	15 (62.5)	14 (58.3)	10 (41.7)
Negative	9 (37.5)	10 (41.7)	12 (50.0)
Indeterminate	0 (0.0)	0 (0.0)	2 (8.3)

Four contacts had unknown exposure degree.

## Discussion

In this study we evaluated the use of IP-10 detection in DPS for LTBI diagnosis after sending the samples via mail. Although, there is a loss in positive results, IP-10 detection in filter paper could be a good alternative as the agreement between IP-10 tests remains strong. Therefore, IP-10 detection in DPS could be of use in TB infection diagnosis when long transportation is required and sample refrigeration is not possible to maintain.

IGRAs have been extensively used to measure T-cell-mediated responses after specific *M. tuberculosis* antigen stimulation. Although their positive predictive value has been characterized as poor, IGRAs have an excellent negative predictive value^6^ and seem to correlate better than TST with the exposure degree to *M. tuberculosis*^7^. During the past few years IP-10 detection has been studied for TB diagnosis both in adults and children as well as immunocompromised patients^9,13,16,23–25^. Regarding the usefulness of IP-10 in children a range of sensitivity (67%-82%) and specificity (87–97%) values which depend on the enrolled cohort characteristics has been described^16,23,24^. Moreover, Ruhwald *et al*. reported an 81% sensitivity and 97% specificity when measuring IP-10 in plasma of adult active TB patients, validating the comparable use of IP-10 with IFN-γ. The overall conclusions from the existing studies are that IP-10 detection seems to be comparable to QFT-G-IT and may add information in young children and immunocompromised patients. Despite the amount of studies on IP-10 performed, fewer assess the use of IP-10 detection in contacts. Wang *et al*. compared IFN-γ and IP-10 levels among active TB patients, household contacts (HHC) and healthy controls, finding that IFN-γ levels correlate with IP-10 and that active TB patients had significantly higher IP-10 levels than the rest followed by HHC and healthy controls^26^. When splitting the HHC into LTBI and uninfected patients, they found that active TB patients remained as the group with higher IP-10 levels but not significantly different from those of LTBI. Biraro *et al*. also compared IP-10 detection in plasma with IFN-γ in a cohort comprising active TB patients and contacts^27^. In contrast to Wang and in agreement with our findings, LTBI patients in their study had the highest production of IP-10 compared to active TB patients and uninfected contacts, and IP-10 levels in active TB patients and LTBI individuals were significantly different compared to those of uninfected contacts.

Unlike IFN-γ detection in DPS which has been considered unreliable^21^, the use of IP-10 in DPS offers the possibility to diagnose TB infection when it is not possible to analyse direct plasma. Using this method, samples do not require a major processing at the clinical setting and they can be kept at room temperature unlike fresh plasma. Therefore, using DPS enables the sending of the sample easily and without damage to the laboratories, which in many cases can be far away from the sampling site. In our previous study, we described as comparable the detection of IP-10 in DPS and in direct plasma with an excellent agreement (κ = 0.91) and with significantly higher levels in active TB patients compared to uninfected controls^18^. To our knowledge, this is the first time that IP-10 detection in DPS has been studied in a contact cohort with different exposure degrees and with more than 50% IP-10 positivity. Tuuminen *et al*.^22^, assessed the performance of IP-10 detection in paediatric contacts, both in direct and dried plasma demonstrating the comparable performance of IFN-γ and IP-10 detection as screening tools with a 100% negative predictive value. The study participants were the two siblings of the index case and 58 classmates. The number of contacts with positive results was very low, with two positive cases at the beginning (one of them was a sibling) and three converters (one of them was the other sibling), thus, no positive predictive value could be established. Our contact cohort comprises a modestly higher amount of positive cases that are contacts form different index cases, the majority TST positive. This study increases the previous evidence that IP-10 detection in direct and dried plasma is comparable to IFN-γ detection in direct plasma in contacts^16^.

Although the amount of IP-10 detected in DPS is lower than the amount detected in direct plasma, therefore causing a decrease of positive results, both IP-10 assays have a strong agreement (κ = 0.77).

Following with the idea of using IP-10 detection in DPS as a potential point-of-care test, IP-10 mRNA detection in dried blood spots (DBS) has also been evaluated in TB patients for this purpose. Blauenfeldt *et al*. demonstrated that, as in IP-10 detection in DPS, IP-10 mRNA is present in significantly higher amount in active TB patients and LTBI individuals compared to uninfected controls (sensitivity 88%, specificity 96%) and that the results are comparable to those obtained in direct blood (sensitivity 85%, specificity 96%)^20^.

Regarding the 5 non-BCG vaccinated contacts with negative QFN-G-IT and IP-10 test results but with a positive TST, we hypothesized, that these could be false-positive TST results due to a non-tuberculous mycobacteria sensitization^28^.

The spread of TB is directly related with degree and duration of the contact exposure with the index TB case, increasing the risk of LTBI infection^29,30^. In order to check whether a closer contact with active TB patients resulted in a higher positivity rate or in the increase of specific cytokines release, contacts were stratified based on time of exposure. In our study, although not statistically significant, those contacts with a higher rate of positive results were those with more than 6 hours of exposure. Moreover, regarding cytokine levels, our results showed no significant differences among groups (*p* > 0.05), therefore in our contact cohort we cannot correlate IP-10 levels with degree exposure to the index case.

Although our overall results support the use of IP-10 detection in DPS for LTBI diagnosis, this study is limited by a relatively small sample size, comprising a total of 80 subjects which may cause an imprecise assessment of the cut-offs. Moreover, there is no gold standard method for LTBI diagnosis, therefore, we cannot truly confirm whether a result is really false positive or negative. Regarding the transportation of the samples, our sending conditions did not seem to interfere in the signal strength nor in the test performance, as we have also reported previously^18^. However, we cannot assure this will be the case in other settings with different transport temperature and timing.

In summary, our study has led to the following conclusions: (i) IP-10 is a useful chemokine that can be used as a biomarker for LTBI; (ii) sending dried plasma in filter paper is a simple process and enables the sending of samples for LTBI diagnosis via mail (iii) IP-10 can be detected in dried plasma by immunological methods; (iv) IP-10 detection in DPS causes a decrease in positive results however, the agreement with IP-10 detection in direct plasma and with QFT-G-IT is high.

The clinical implication of this study is the development of a new procedure for LTBI diagnosis in contacts that simplifies the sending of previously stimulated samples from the collection site to the analysis site without sample damage using simple protocols. Having a simple and safe method to send samples through mail with no need for refrigeration enables LTBI diagnosis in faraway settings, thus setting us a step closer to improving LTBI diagnosis and disease control.

## Material and Methods

### Study setting and patient recruitment

In this retrospective case-control study we included whole blood samples from microbiologically confirmed active pulmonary TB patients and individuals from contact tracing studies (contacts of smear positive patients). In order to correlate the amount of cytokine detected with the time of exposure, contacts were divided into three subgroups regarding time of exposure to active TB patients: (i) more than 6 hours of exposure per day, (ii) less than 6 hours of exposure per day, and (iii) sporadic contact (not daily). Moreover, and aware of the lack of gold standard, contacts were divided into two groups regarding the QFN-G-IT result. Those with a negative test result were considered as uninfected controls and those with positive results were considered as LTBI individuals.

Such samples were collected at two medical centers in Barcelona, Spain: Unitat de Tuberculosi de Drassanes - Hospital Universitari Vall d’Hebron and Unidad Clínica de Tratamiento Directamente Observado “Serveis Clinics”.

The study was approved by the Ethics Committee Hospital Universitari Germans Trias i Pujol (http://www.ceicgermanstrias.cat/). This research was performed in accordance with the relevant guidelines/regulations. Written informed consent was collected from each subject before blood sampling and a detailed questionnaire with the following variables was filled in: age, gender, country of birth, smear and culture result, tuberculin skin test result, BCG vaccination and time of exposure.

### Tuberculin skin test

To perform the tuberculin skin test (TST) we followed the Spanish guidelines^31^ using the Mantoux method with 2-TU of PPD RT23 (Statens Serum Institut, Copenhagen, Denmark). Briefly, 0.1 ml of PPD solution was injected intradermally by experienced personnel in the patients’ forearm^31^. The induration diameter was measured after 48–72 hours. When the induration had a diameter equal to 5 mm or above the test was considered positive regardless BCG vaccination status.

### IFN-γ detection in direct plasma

To detect IFN-γ production, we used the QuantiFERON-TB Gold In-Tube (QFN-G-IT; Qiagen, Düsseldorf, Germany) procedure following manufacturer’s instructions. Briefly, from each patient 3 ml of blood (1 ml per tube) were extracted. The reactivity obtained in the nil tube was subtracted from the IFN-γ value of the antigen (RD1 antigens cocktail: ESAT-6, CFP-10, and TB7.7) and mitogen tubes. An IFN-γ level equal or above 0.35 IU/ml after antigen stimulation is recorded as a positive result. If the stimulation was negative and the value of the positive control was less than 0.5 IU/ml or the negative control higher than 8.0 IU/ml the result was considered indeterminate. Throughout this study, the amount of IFN-γ will be shown in pg/ml to ease the comparison with IP-10 (1 IU/ml = 50 pg/ml, National Institute for Biological Standards and Control, [NIBSC], UK).

### IP-10 detection in direct plasma

IP-10 was detected in the previously QFN-G-IT analysed plasma using a non-commercial in-house ELISA^18^. Briefly, 30 time diluted plasma samples were incubated for 2 hours in an ELISA plate pre-coated with specific IP-10 monoclonal antibodies. Plate was washed and TMB substrate was added. After incubating for 30 minutes, the reaction was stopped with H_2_SO_4,_ and the absorbance was read (450nm-630nm)^21^. The nil stimulated sample response was subtracted from that of the antigen and mitogen stimulated samples.

### IP-10 detection in dried plasma spots

At the medical centres, two plasma drops of 25 µl from each sample were dried in Whatman903 filter paper for 3–4 hours at room temperature (RT), away from direct sunlight. After 5 days at RT, the samples were sent by postal service to the lab in a plastic bag with desiccants, to avoid humidity. Once in the lab, after a 2-day transport, samples were stored at RT and IP-10 detection analyses were performed within 7 days after arrival. The cytokine detection was done using a non-commercial in-house ELISA and following the procedure previously described^21^. Briefly, each dried plasma spot (DPS) was punched by a standard office puncher of a 5 mm diameter. The two discs from each sample were placed into the same well of a 96 well plate with dilution buffer. Conjugate buffer was added and the sample was mixed. After 2 hours of incubation at RT and dark conditions, the wells were washed and the discs were knocked out. In order to measure IP-10 levels, TMB stabilized chromogen was added and after 30 min in the dark the reaction was stopped. The plates were read at 450–630 nm.

### Statistical analysis

The correlation between IP-10 detection in direct plasma and dried plasma, was assessed using Spearman correlation coefficient for a stability overview approach and to determine whether IP-10 levels detected by each test were comparable. In order to study the agreement among the different tests and assess the interrater reliability, we used Cohen’s kappa (κ) coefficient, excluding indeterminate results. Kappa values range from 0 (agreement that could be expected from chance) to +1 (perfect agreement)^32^. Agreement was considered strong, intermediate or weak, when kappa values were above 0.60, between 0.60 and 0.40, or below 0.40, respectively. We used Mann-Whitney U test, two tailed, to compare the amount of both cytokines released in each QFN-G-IT tube. We considered as significant *p* values those under 0.05.

In order to establish positivity cut-offs for both IP-10 detection tests, we performed a receiver operating characteristic (ROC) curve analysis. Due to the well-known non-specificity of the TST, we have considered QFN-G-IT positive contacts as LTBI patients and QFN-G-IT negative contacts as uninfected controls. We chose positivity cut-offs for IP-10 detection that gave a high specificity without losing sensitivity. As indeterminate result cut-offs, we chose the lower value of mitogen after nil subtraction of those negative QFT-G-IT samples.

Analysis and graphs were performed using SPSS statistical software (SPSS version 15.0; SPSS Inc, Chicago, IL, USA) and GraphPad Prism version 5.00 (GraphPad Prism version 5.00 for Windows, GraphPad Software, San Diego California USA, www.graphpad.com).

## Data Availability

Due to participant privacy, the data collected for this study is available upon request to the corresponding author.
